# Supplementary material for: Ultrastrong and ductile steel welds achieved by fine interlocking microstructures with film-like retained austenite
Source: Nat Commun. 2024 Feb 12;15:1301. doi: 10.1038/s41467-024-45470-1 (PMC10861522; doi:10.1038/s41467-024-45470-1)
Supplement: Supplementary file 1 — Supplementary information [file 41467_2024_45470_MOESM1_ESM.pdf]

Supplementary Information

Ultrastrong and ductile steel welds achieved by fine interlocking microstructures with film-like retained austenite

Joonoh Moon [1,*], Gyuyeol Bae [2,*], Bo-Young Jeong [2], Chansun Shin [3], Min-Ji Kwon [1], Dong-Ik Kim [4], Dong-Jun Choi [4], Bong Ho Lee [5], Chang-Hoon Lee [6], Hyun-Uk Hong [1], Dong-Woo Suh [7], Dirk Ponge [8]

[1] Department of Materials Convergence and System Engineering, Changwon National University, 20 Changwondaehak-ro, Changwon, Gyeongnam, 51140, Republic of Korea

[2] Steel Solution Research Lab., Technical Research Lab., POSCO, 100 Songdogwahak-ro, Yeonsu-gu, Incheon 21985, Republic of Korea

[3] Department of Materials Science and Engineering, Myongji University, Yongin 17058, Republic of Korea

[4] Energy Materials Research Center, Korea Institute of Science and Technology, Hwarang-ro 14-gil 5, Seongbuk-gu, Seoul 02792, Republic of Korea

[5] Advanced Analysis Team, Inst. of Next-Generation Semicond. Convergence Technol., Daegu Gyeongbuk Institute of Science and Technology, 333, Technojungang-daero, Hyeonpung-eup, Dalseong-gun, Daegu 42988, Republic of Korea

[6] Steel Department, Korea Institute of Materials Science, 797 Changwon-daero, Seongsan-gu, Changwon 51508, Republic of Korea

[7] Graduate Institute of Ferrous & Energy Materials Technology, Pohang University of Science and Technology, 77 Cheongam-ro, Nam-gu, Pohang, Gyeongbuk 790-784, Republic of Korea

[8] Max-Planck-Institut für Eisenforschung, Max-Planck-Straβe 1, Düsseldorf 40237, Germany

* <u>Correspondence to:</u> mjo99@changwon.ac.kr, gbae@posco.com

These authors jointly supervised this work: Joonoh Moon, Gyuyeol Bae

This file includes:

Supplementary Table 1

Supplementary Fig. 1 – 3

Supplementary Table 1. Welding parameters

GIGA steel weld		Welding parameters			
		Current, A	Voltage, V	Travel speed, cm/min	Heat input, kJ/cm
1.2GPa H-Nb *(1.2GPa H-Nb/L-Ni)*	Developed	127	22	80	1.7
1.2GPa H-Ni *(1.2GPa H-Ni/L-Nb)*	Conventional	124	23	80	
1.0GPa H-Nb *(1.0GPa H-Nb/L-Ni)*	Developed	161	24	80	2.3
1.0GPa H-Ni *(1.0GPa H-Ni/L-Nb)*	Conventional	163	24	80	

* Arc efficiency of MAG welding for calculating heat input : 0.8

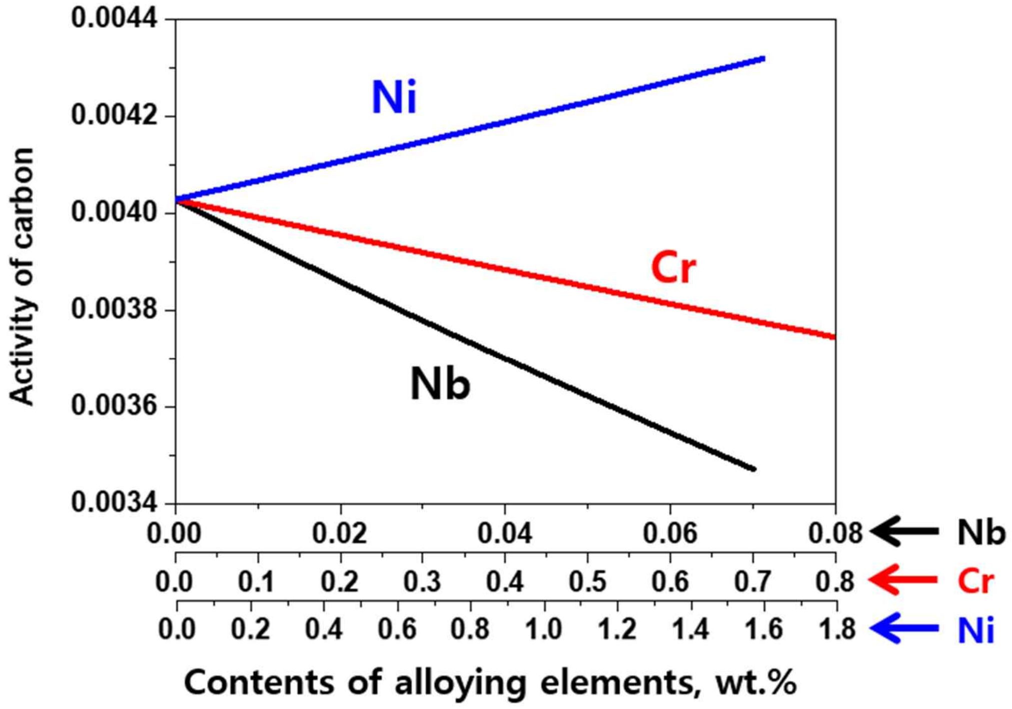

Supplementary Figure 1. Variation in carbon activity with increasing Nb, Cr, and Ni contents in the same composition with 1.0 GPa steel welds calculated by Thermo-Calc. software (TCFE 12 database)

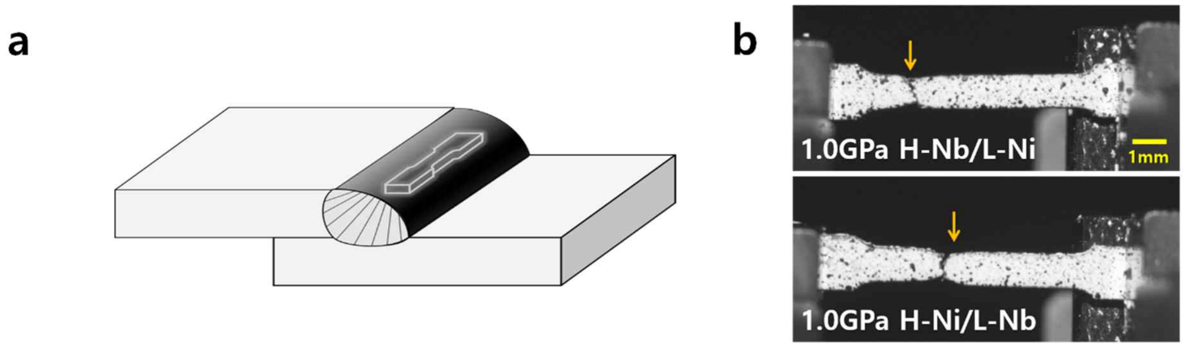

Supplementary Figure 2. Schematics of the micro-tensile test. a Specimen prepared from the weld. **b** Snapshot images of the fractured samples.

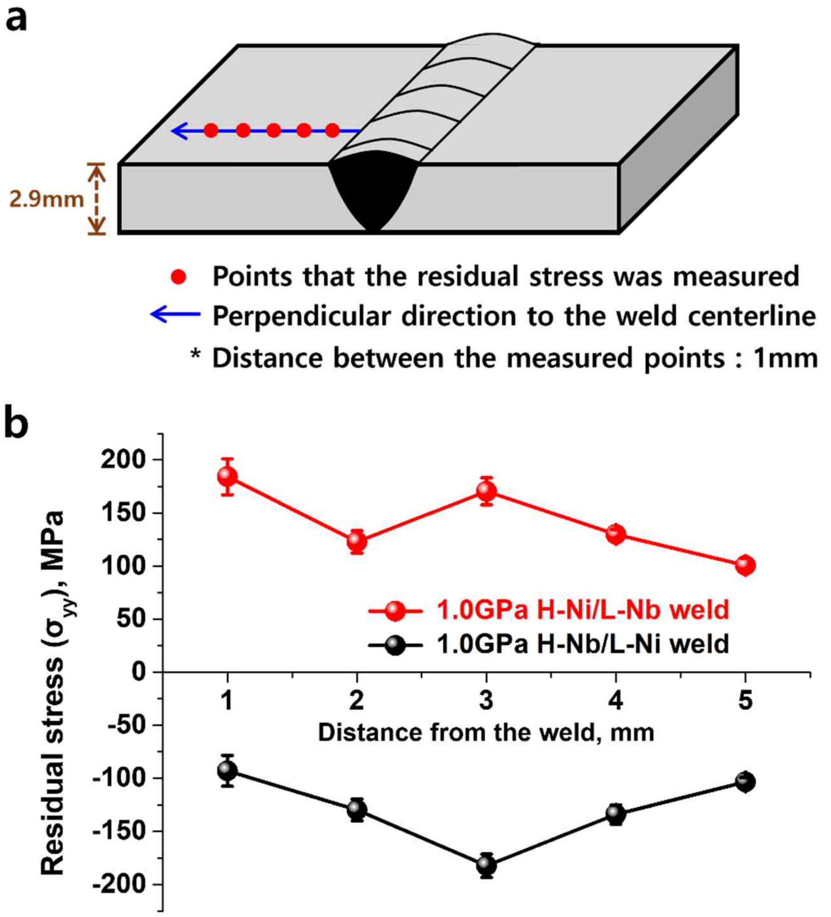

Supplementary Figure 3. Residual stress measurement around the welds. a Schematic diagram showing the points that residual stress was measured in butt weld. **b** Residual stress distribution around the welds. All the error bars in **(b)** represent the standard deviation (n=3 independent experiments).
